# Wide‐Range Adaptive Metal Oxide for Hydrogen Sulfide Detection From Earth to Space‐Like Environments

**DOI:** 10.1002/advs.202515684

**Published:** 2026-02-08

**Authors:** Xi Chen, Jiaxin Chen, Yanghui Liu, Junhao Cao, Yang Yuan, Kaixin Chen, Yilang Ye, Tongshuai Yang, Yongqing Fu, Wei Luo

**Affiliations:** ^1^ School of Integrated Circuits Huazhong University of Science and Technology Wuhan P. R. China; ^2^ School of Instrument Science and Technology Xi'an Jiaotong University Xi'an China; ^3^ Research Institute of Huazhong University of Science and Technology in Shenzhen Shenzhen P. R. China

**Keywords:** gas sensors, H_2_S detection, metal oxide semiconductors (SMOs), space sensing, surface–bulk–interface engineering

## Abstract

Chemiresistive gas sensors based on semiconducting metal oxides for toxic gas detection are widely explored for terrestrial applications under ambient environments, but their potential in extraterrestrial applications remains underexplored. Herein, we developed porous Cu‐doped SnO_2_ microspheres, enabling high sensitivity and selectivity toward hydrogen sulfide (H_2_S), from the ambient air (25°C, 10^5^ Pa) to extreme conditions (−40°C, ∼10^−^^4^ Pa) designed to simulate the space‐like oxygen defects and cryogenic environments. Hierarchical porosity enables efficient gas diffusion across pressure regimes, and Cu^2^^+^ doping and oxygen vacancies thus enable oxygen‐independent chemisorption. Moreover, in situ‐formed chemical adsorption promotes interfacial charge transfer, which exhibits partial reversibility. The semi‐quantitative framework represented by a CuS kinetic proxy, combining numerical simulations based on Wolkenstein adsorption theory, finite element methods, and experimental results, reveals a dual‐mechanism paradigm. At ambient conditions, the oxygen‐adsorption‐driven redox reaction is dominant. In contrast, under a vacuum around 10^−4^ Pa, direct chemisorption and interfacial charge transfer primarily govern the gas adsorption responses. This study offers a generalized metal‐oxide platform for gas detection for future space exploration and life‐support monitoring systems.

## Graphical abstract

This work presents a gas sensing platform based on Cu‐doped SnO_2_ microspheres that operates reliably from ambient to space‐like conditions. The sensor maintains performance across vacuum and cryogenic environments, bypassing traditional oxygen‐mediated mechanisms, utilizing direct chemisorption and interfacial charge transfer. Simulations and experiments show their adaptive sensing behavior under harsh conditions.

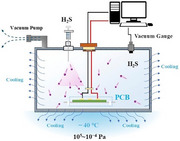

## 1 Introduction

Hydrogen sulfide (H_2_S) [1, 2] is a key biomarker for biological activities and a planetary indicator [3, 4], its reliable trace detection is essential for environmental monitoring life‐support diagnostics, and safety assurance [5]. On Earth, H_2_S is one of the most toxic and corrosive gases widely released from petroleum refining, wastewater treatment, geothermal activity, and volcanic eruptions, posing severe environmental and occupational hazards [2]. In extraterrestrial environments (<10^−^^6^ Pa, <100 K), sulfur‐bearing volatiles are reported across a wide range of settings. Gaseous H_2_S is observed at a mole fraction of 0.4–0.8 ppm levels in the upper clouds of Uranus [6, 7],∼1–3 ppm at cloud tops on Neptune [8], and upper limit of 330 ppb (at 0.27 mbar) H_2_S in Titan's atmosphere [9]. The lunar LCROSS plume showed H_2_S as the second most abundant detected volatile after H_2_O [10]. Moreover, its strong reducing nature and high electron‐donating capability make it an excellent probe molecule to study gas–solid interactions and charge‐transfer mechanisms under oxygen‐deficient and cryogenic conditions. Accordingly, the detection of H_2_S from ambient environments to high vacuum and cryogenic regimes is crucial for future applications.

Chemiresistive gas sensors based on semiconductor metal oxides (SMOs) [11, 12, 13] have dominated terrestrial applications [14] owing to their high sensitivity, low cost, and compatibility at ambient conditions [15, 16]. However, most commercial sensors are designed for the specific or narrowly defined environmental conditions, such as high pressure (GPa), high temperature (> 300°C), or high humidity [17]. Besides, most current gas‐sensing technologies are limited to terrestrial applications, such as industrial safety or health monitoring systems, and commonly lack the adaptability required for continuous deployment in various harsh environments. Their functionality often depends on oxygen‐mediated redox reactions, where surface‐adsorbed oxygen species serve as charge‐transfer intermediates. In this work, we focus on extending the sensing functionality toward space‐like environments characterized by low oxygen partial pressure and subzero temperature.

In recent decades, researchers have explored various engineering strategies to extend the operational range of SMOs based [15, 18, 19] sensors. Surface engineering, particularly micro‐ and mesoporous architectures, enhances gas accessibility and reaction kinetics by increasing active surface areas and facilitating efficient diffusion [20]. Bulk engineering, such as introducing oxygen vacancies or dopants, has been commonly applied to modulate charge carrier density and adsorption energetics, thereby enabling gas responses at lower temperatures [21, 22]. In parallel, interfacial engineering, e.g., via *p*–*n*, *n*–*n*, or Schottky heterojunctions, has provided additional channels for gas‐induced charge redistribution, in some cases enabling sensing under oxygen‐deficient conditions. Despite these advances, most studies have focused on isolated extreme conditions, either low temperature or vacuum. For example, our previous work has demonstrated that Sb‐SnO_2_/g‐C_3_N_4_ under ultraviolet light (365 nm) at −40°C enables 1 ppm_a_ H_2_S detection [23]. Chakraborty and Mondal also achieved sub‐zero (−78°C) chemiresistive NH_3_ sensing using Sb‐SnO_2_/polypyrrole hybrids [24]. CeO_2_‐based gas sensor showed pressure‐independent resistance behavior governed by H_2_/O_2_ partial pressure ratios under a vacuum condition, enabling reliable detection down to 10^−^^5^ Pa [25]. However, these approaches typically operate within isolated environmental domains, and few have addressed the complex coupling among temperature, pressure, and gas composition. Moreover, the competition between oxygen adsorption and direct gas–solid chemisorption mechanisms, and how this interplay evolves across environmental transitions, have not been comprehensively explored. In this study, the experiments were performed down to ∼10^−^^4^ Pa and −40°C, which represent a practically attainable window where oxygen adsorption becomes negligible [26]. Such conditions provide a realistic and scientifically meaningful proxy for oxygen‐free atmospheres characteristic of extraterrestrial environments.

**FIGURE 1:**
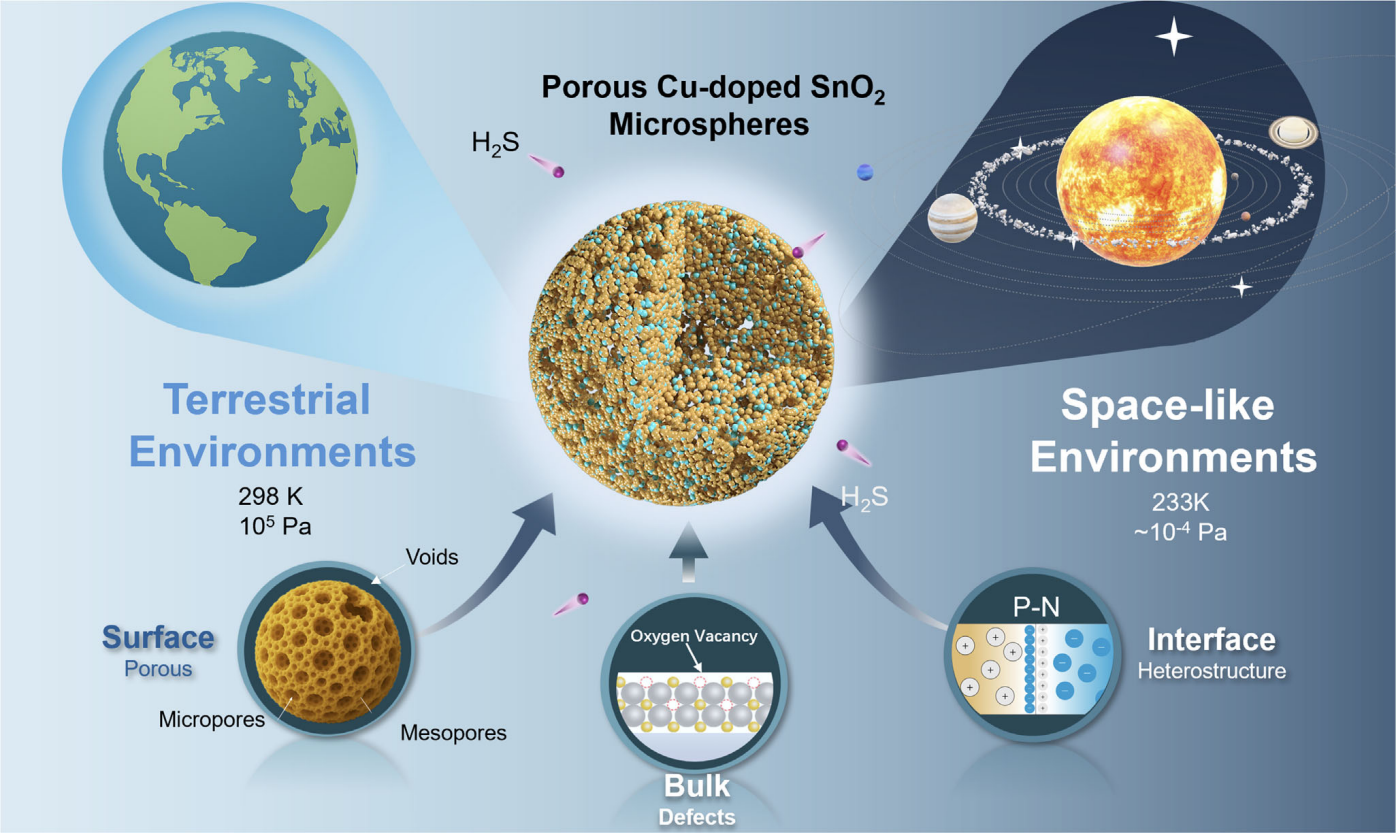
Scheme for the gas detection H_2_S gas sensing via “Surface–Bulk–Interface” engineering under space‐like environments.

Herein, we propose a conceptual framework that systematically integrates three well‐established engineering strategies, i.e., surface, bulk, and interfacial engineering, to develop a gas sensor platform capable of operating from Earth into space‐like environments. As a demonstration example, by developing porous Cu‐doped SnO_2_ microspheres (CSMs), we achieved H_2_S gas detection in atmospheric and vacuum conditions. The incorporation of Cu^2^^+^ dopants effectively reduces the activation energy for H_2_S dissociation, whereas oxygen vacancies [27] and interconnected cavities enhance the chemisorption capacity. Furthermore, the in situ formation of CuO/SnO_2_ heterojunctions facilitates favorable band alignment and efficient interfacial charge separation (as shown in Figure 1). The resulting sensors exhibit reliable performance across a wide environmental spectrum, from atmospheric pressure (10^5^ Pa) to high vacuum (10^−^^4^ Pa), and from room temperature down to −40°C. This work establishes a proof‐of‐concept framework that bypasses traditional oxygen‐mediated sensing mechanisms, achieving functionality under oxygen‐deficient, cryogenic, and low‐pressure conditions. Beyond its extreme‐environment capability, the platform remains compatible with standard earth‐based applications, offering a strategy for next‐generation gas sensors spanning both terrestrial and extraterrestrial domains.

## 2 Results

### 2.1 Design of CSMs From Earth to simulated space environments

Space detection missions invariably involve highly dynamic environmental transitions, where atmospheric pressure progressively drops to vacuum or even ultra‐high vacuum levels, accompanied by sharp temperature declines to subzero or cryogenic regimes (as illustrated in Figure 1). Although strategies have already demonstrated effectiveness in enhancing SMO gas sensing under specific terrestrial conditions [28], their performance under low‐temperature and low‐pressure regimes remains unexplored. We systematically integrated established tuning strategies to investigate the operational boundaries of SMO‐based gas sensors. Hierarchical porosity facilitates gas transport, bulk‐level doping with oxygen vacancy engineering enhances charge conductivity [29, 30], and in situ heterojunctions enable oxygen‐independent sensing. All these three together ensure a stable performance across variable oxygen availability, pressure, and temperature conditions. For simulating the extreme outer space conditions, we designed and fabricated a bespoke chamber which achieved a base pressure (from 10^5^ and down to 10^−^^4^ Pa) and a wide temperature range from room temperature down to −40°C. The details are provided in Figure S1.

### 2.2 Characteristics of Gas Sensing of CSMs

**FIGURE 2:**
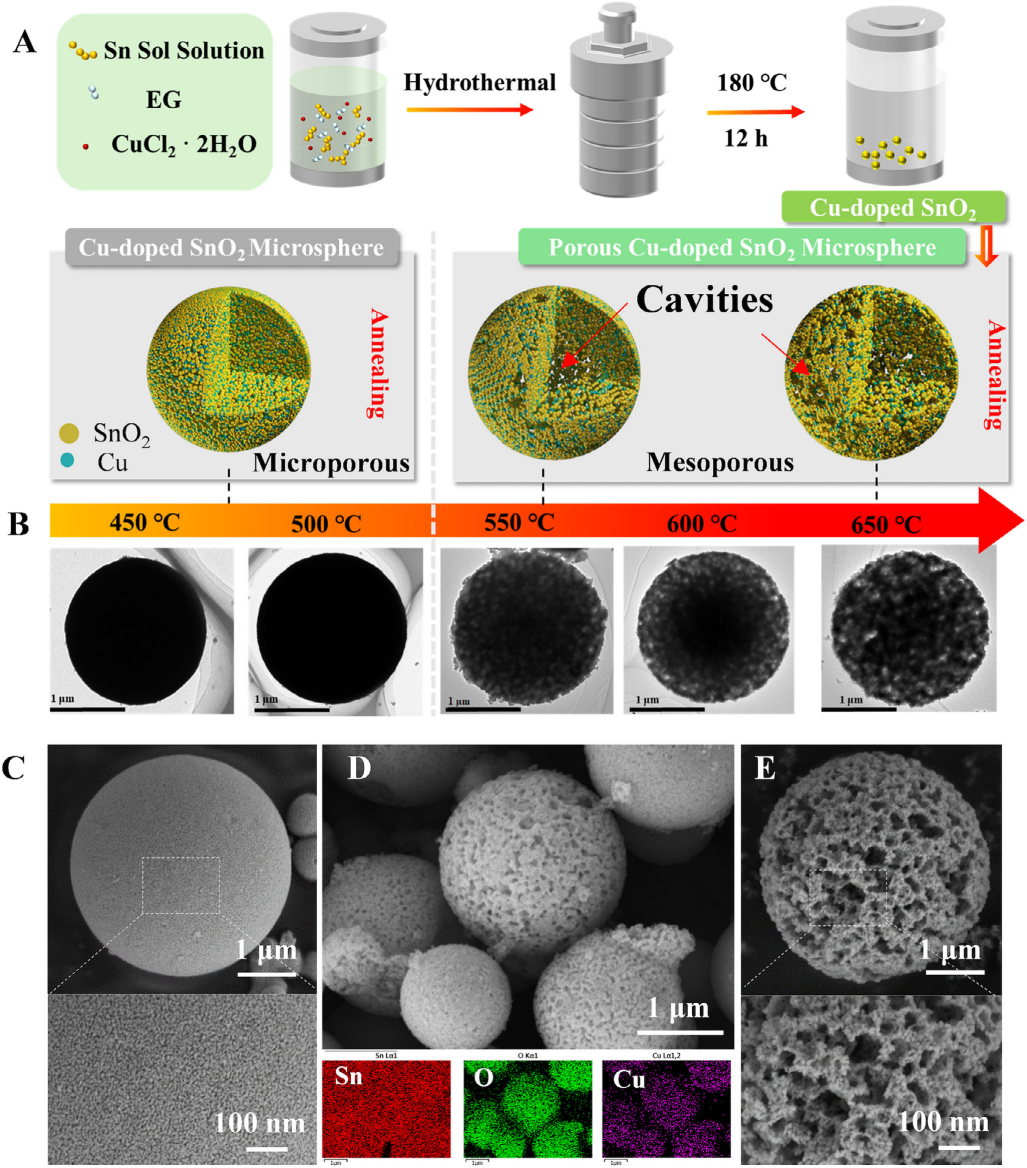
Annealing‐induced pores formation in Cu‐doped SnO_2_ microspheres. (A) Scheme of the synthesis process and temperature‐dependent porous microspheres. (B) TEM images of microspheres annealed at different temperatures (450°C–650°C) for 1 h. (C–E) SEM images (450°C, 550°C, 650°C) and EDS elemental mapping of Cu‐doped SnO_2_.

The CSM was synthesized by a simple hydrothermal process followed by post‐annealing (Figure 2A). CuCl_2_·2H_2_O was added into the Sn sol‐solution with ethylene glycol (EG), with a solvothermal synthesis process to obtain dense Cu‐doped SnO_2_ microsphere precursors. Subsequent annealing at varied temperatures induced structural transformations. Transmission electron microscope (TEM) images in Figure 2B show the evolution of internal macroscopic cavities within the microspheres as the annealing temperature increases from 450°C to 650°C. At temperatures below 500°C, the microspheres remain relatively dense, whereas annealing above ∼550°C leads to the formation of larger internal cavities due to thermal shrinkage and grain coalescence. It is important to note that these TEM‐visible cavities—typically hundreds of nanometers in size—are distinct from the 8–20 nm mesopores quantified by Brunauer–Emmett–Teller (BET) analysis. The latter reflect nanoscale porosity within primary grains, whereas the former represent macroscopic structural features that do not contribute significantly to the measured BET surface area.

Figure 2C–E show the surface morphologies obtained from the scanning electron microscopy (SEM), which demonstrate the transformation from smooth, compact spheres to porous architectures. Figure 2C–E show the distribution of Sn, O, and Cu elements across the porous framework, obtained using the energy‐dispersive X‐ray spectroscopy (EDS) mapping. The continuous and relatively uniform distribution of the Sn signal, in contrast to the localized Cu and O mappings, is attributed to the high X‐ray yield of the matrix element and the cumulative signal contribution from the underlying stacked CSM spheres; this phenomenon further confirms the formation of a well‐interconnected sensing network that facilitates charge transport. The annealing‐guided engineering method offers a good pathway for tuning gas diffusion, enhancing surface accessibility, and improving catalytic activity in those doped metal oxide nanostructures. Figure S2 and Table S1 show the BET surface area results, which were significantly decreased from 59.3 m^2^/g (CSM‐450) to 17.9 m^2^/g (CSM‐650), accompanied by a reduction in monolayer adsorption volume from 13.6 to 4.1 cm^3^/g.

In contrast, the average pore size only increased from 8.2 to 19.5 nm, suggesting that the moderate enlargement of mesoporous features was predominantly driven by thermally induced pore coalescence and grain growth. Notably, the adsorption constant (C) increased significantly from 75.636 for CSM‐450 to 260.99 for CSM‐650, indicating that the enhanced adsorbent–adsorbate interactions can be attributed to the increased crystallinity or defect‐driven active sites. It should be emphasized that these nanoscale mesopores, quantified by BET analysis, are distinct from the cavities’ regions (> 500 nm) observed in SEM images (Figure 2B), which correspond to macroscopic cavities between aggregated grains or inside the microspheres. The results primarily arise from the Kirkendall effect and grain coalescence during annealing, rather than the evolution of intrinsic mesoporosity [31]. Consequently, despite the reduced specific surface area, the thermally induced mesoporous framework and macroscopic porous network collectively facilitate gas diffusion and reaction kinetics across multiple length scales.

**FIGURE 3:**
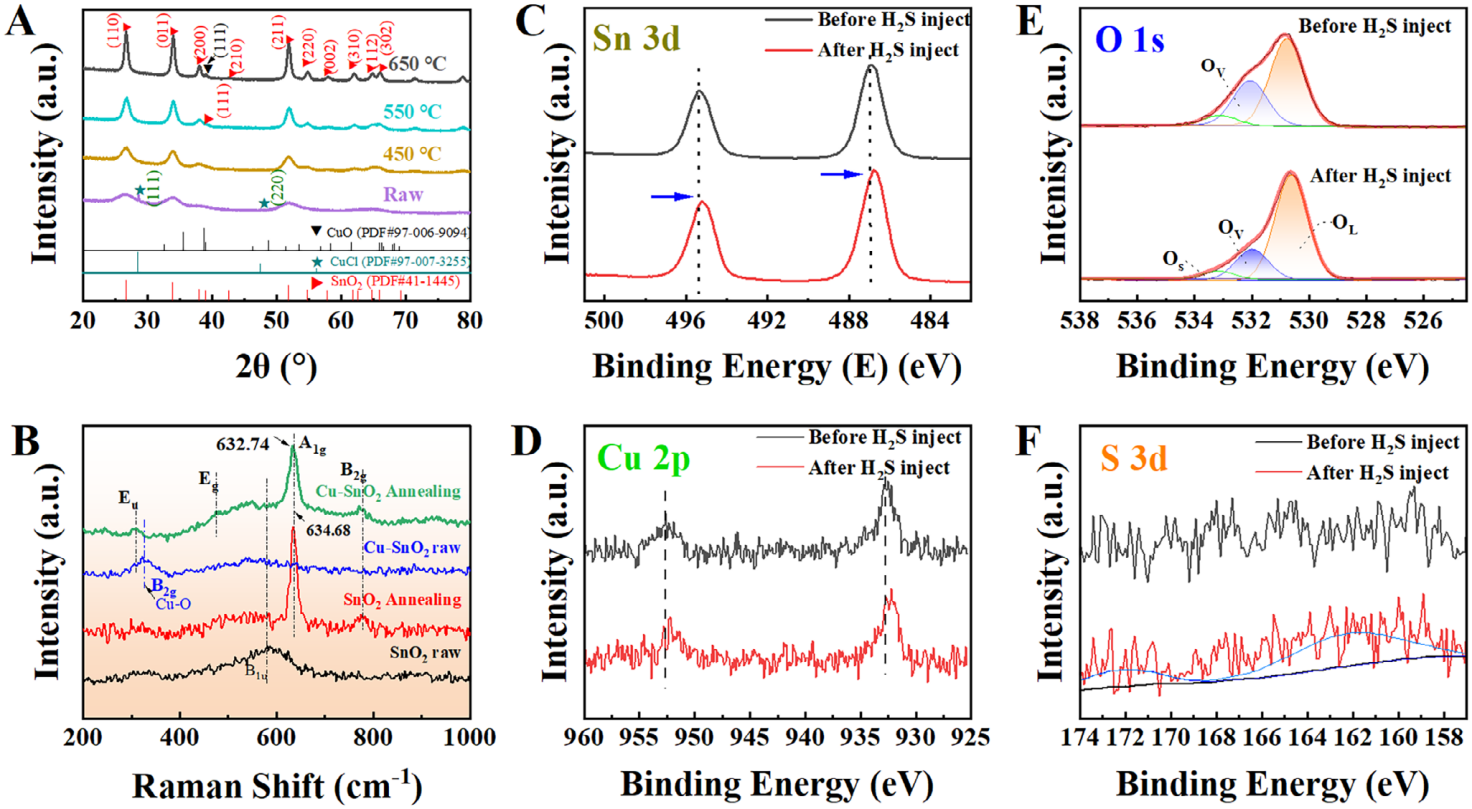
Structural and surface chemical analysis of Cu‐doped SnO_2_ microspheres. (A) XRD patterns of raw Cu‐doped SnO_2_ and CSM‐450, CSM‐550, CSM‐650. (B) Raman spectra of the raw and annealed CSM‐650 sample. (C–F) XPS spectra of (C) Sn 3d, (D) Cu 2p, (E) O 1s, and (F) S 3d before and after H_2_S exposure by CSM‐550.

Structures and surface chemical evolution of Cu‐doped SnO_2_ microspheres are shown in Figure 3 and Figure S3, revealing crystallization of SnO_2_ and the incorporation of Cu‐related species. X‐ray diffraction (XRD) patterns (Figure 3A) reveal diffraction peaks corresponding to the (110), (101), (211), and (301) planes of tetragonal rutile SnO_2_ (JCPDS #41‐1445), confirming successful crystallization after annealing. In the precursor samples, additional peaks associated with CuCl phases are present, arising from redox reactions during hydrothermal synthesis. After post‐synthesis in a muffle furnace, the CuCl‐related signals  diminished, revealing thermal decomposition or solid‐state transformation. Such crystallization and phase transition of the Cu–SnO_2_ system were progressive as the annealing temperatures were changed from 450°C to 650°C. Raman spectroscopy analysis (Figure 3B) confirmed the structural evolution of SnO_2_ during the hydrothermal synthesis and subsequent annealing. All the calcined samples exhibited the characteristic rutile SnO_2_ modes of A_1g_ (∼638 cm^−^^1^) and B_2g_ (∼780 cm^−^^1^), corresponding to its symmetric and asymmetric Sn–O stretching vibrations, respectively [32, 33]. In contrast, the as‐synthesized SnO_2_ precursor displayed a broad and irregular B_1u_ mode (∼570.4 cm^−1^), indicative of its poor crystallinity and nanometric grain size. This peak broadening mainly originated from surface defect formation and lattice distortion introduced during the hydrothermal growth. The Cu‐doped precursors exhibited an additional B_2g_ peak at ∼239 cm^−1^ and attenuated SnO_2_ signals, suggesting formation of surface‐dispersed Cu^2+^ species at this stage. After annealing, the CSM samples showed a blue shift (∼1.9 cm^−^^1^) and broadening of the A_1g_ peak, reflecting the lattice expansion caused by Cu^2+^ (0.73 Å) substitution of Sn^4+^ (0.69 Å).

X‐ray photoelectron spectroscopy (XPS) analysis was applied to investigate the chemical states of CSMs before and after H_2_S exposure (Figure 3C–F). In addition, both the Sn 3d and Cu 2p spectra (Figure 3C,D) exhibit red shifts (0.1 eV) upon H_2_S exposure, revealing the occurrence of surface redox reactions and strong analyte–sensor interactions. In Figure 3E, the deconvoluted O 1s spectrum shows a peak at ∼532 eV, attributed to surface oxygen vacancies (OVs) of CSM‐550, which decreased after exposure to H_2_S gas [27, 34]. This decline reveals that the OVs were actively involved in the redox reaction with H_2_S, strongly confirming their role as reactive sites for gas adsorption and sensing. Furthermore, the appearance of S 3d peaks (Figure 3F) after H_2_S injection confirmed that gas adsorption and partial chemical reactions occurred on the sensor surface, thereby revealing the active role of engineered surface states in sensing performance. Because the XPS samples were sealed in a 1% H_2_S/N_2_ bag and subsequently pumped down to the XPS analysis pressure (10^−7^–10^−^^8^ Pa), weakly physisosorbed species are expected to desorb completely. Therefore, the observed signals mainly reflect strongly chemisorbed Cu/Sn–S species, which are responsible for the high selectivity. Owing to the high chemical affinity of copper, the interfacial species likely manifest as disordered or non‐stoichiometric Cu_x_S clusters rather than a bulk crystalline phase, serving as the essential chemical trigger for the sensor response.

### 2.3 Resistance Modulation Governed by Surface Oxygen Desorption in Vacuum

**FIGURE 4:**
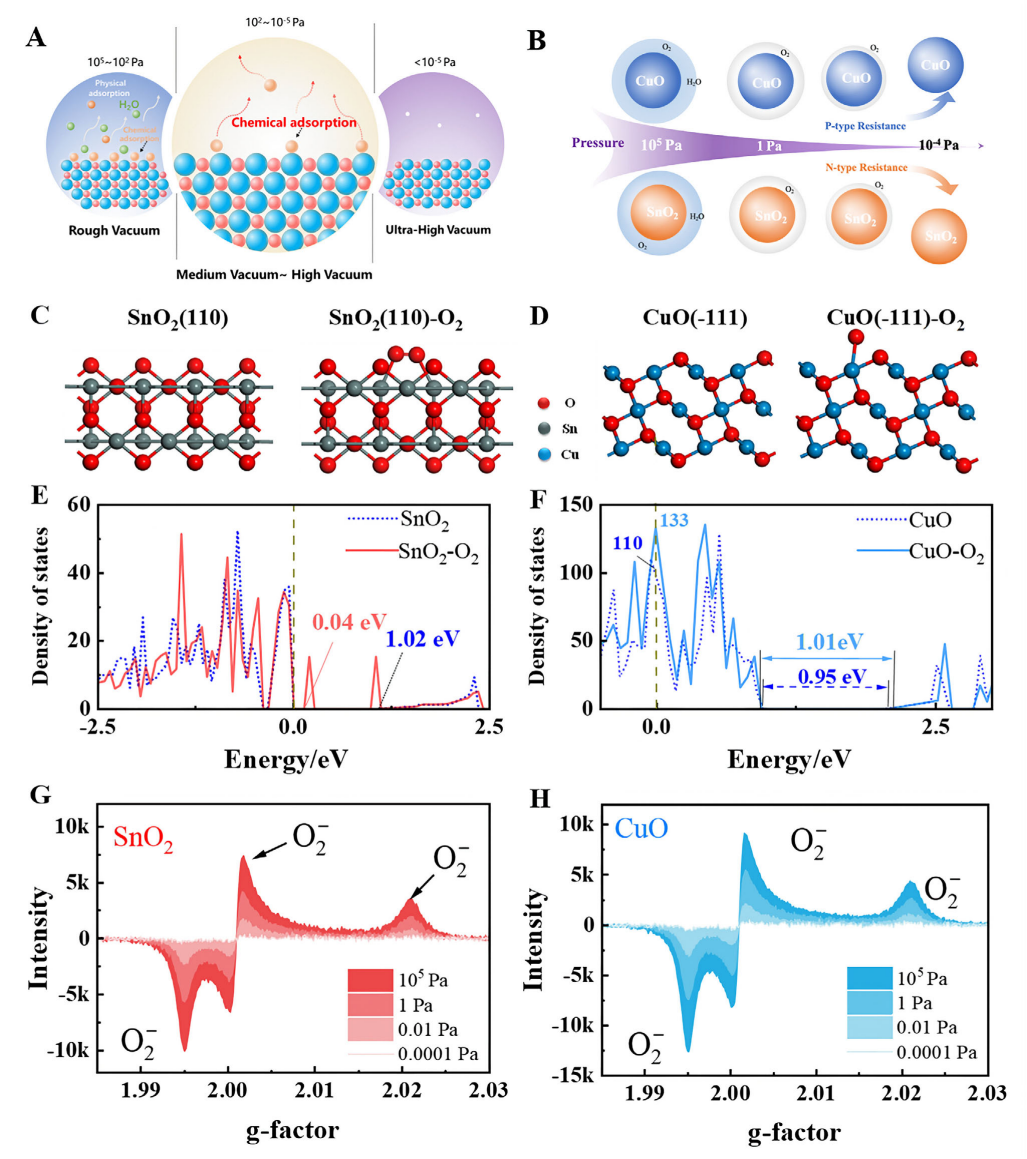
The pressure‐dependent oxygen adsorption behavior of semiconductors. (A) and (B) Schematic illustrations of the adsorption behavior of p‐type and n‐type semiconductors across ambient to vacuum conditions. Structures of SnO_2_ (C) and CuO (D) before/after oxygen adsorption. Density of states (DOS) of SnO_2_ (E) and CuO (F) before/after oxygen adsorption. The intensity changes of the electron paramagnetic resonance (EPR) signal of O_2_ on SnO_2_ (G) and CuO (H) under different pressures at 298 K.

The chemical adsorption of O_2_ molecules induces significant structural rearrangements of SMOs on surfaces [35], leading to notable changes in their electronic properties (Figure 4A,B). We employed density functional theory (DFT) to explore how oxygen adsorption influences semiconductor surfaces' electronic structure and conductivity. In our model, the “depletion regime” should be understood as an effective electronic state under high‐vacuum conditions, where oxygen‐related surface charge dominates, and other weakly bound species (e.g., N_2_, H_2_O) are negligible.

The model does not imply that SnO_2_ remains in depletion across all pressures. Instead, it describes the intrinsic sensing regime that emerges only when the surface is stripped of adsorbed oxygen. The atomic structures of SnO_2_ (110) and CuO (–111) surfaces before and after single O_2_ adsorption are shown in Figure 4C–F, respectively. For the pristine SnO_2_(110) surface, density of states (DOS) calculations reveal a clear semiconducting character with a bandgap of approximately 1.02 eV between the valence band maximum (VBM) and conduction band minimum (CBM). Following single O_2_ chemisorption, distinct mid‐gap states emerge, effectively narrowing the free carrier population by acting as trap states to 0.98 eV. This electronic restructuring implies a reduction in conductivity due to carrier localization. In parallel, the CuO (–111) surface exhibits a significant shift in its DOS upon O_2_ adsorption, with the primary electronic contribution near the Fermi level shifted from 110 to 133 and the bandgap widened from 0.95 to 1.01 eV. Besides, according to the Boltzmann transport theory, the conductivity of pristine SnO_2_ (110) surface at 100 K is 1.89 × 10^6 ^S/m and remains a similar value at 298.15 K, indicating negligible temperature dependence (Figure S4). However, for the SnO_2_ (110) surface with the adsorbed O_2_, the conductivity at 100 K is 1.42 × 10^6^ S/m and slightly decreases to 1.41 × 10^6^ S/m at 298.15 K. The conductivity of SnO_2_ (110) decreases by approximately 0.47–0.48 × 10^6^ S/m. In contrast, the conductivity of the raw CuO (–111) is 2.27 × 10^5^ S/m at 100 K and slightly increases to 2.28 × 10^5^ S/m at 298.15 K. For the CuO (–111) surface with adsorbed O_2_, the conductivity is 2.38 × 10^5^ S/m at 100 K and increases slightly to 2.40 × 10^5^ S/m at 298.15 K. This trend suggests that oxygen adsorption suppresses carrier mobility and introduces weak temperature‐dependent conductivity degradation due to the formation of localized states and enhanced carrier scattering.

We further conducted EPR tests to confirm this phenomenon, as shown in Figure 4G,H. As the pressure decreases, the intensity of O_2_^−^ signals diminishes significantly, indicating a reduced concentration of adsorbed oxygen. The same trend was observed in SnO_2_ and CuO. When the pressure is changed to 10^−4^ Pa, the O_2_^−^ signal becomes nearly undetectable, indicating that surface‐adsorbed oxygen is effectively depleted under ultra‐high vacuum conditions. This serves as the primary explanation for the observed resistance changes, as supported by numerical simulations (NS) based on the Wolkenstein adsorption theory, which accounts for the chemical adsorption of O_2_ via the surface potential (*V*_s_). Figures S5–S7, supported by Tables S1 and S4, show the conductivity changes due to O_2_ chemisorption. The observed resistance variations in CSMs can be attributed to the coupling behavior between p‐type and n‐type semiconductor characteristics induced by different Cu doping levels. The calculated *V*_s_ and conductivity variation of SnO_2_ were implemented as material functions in a COMSOL reaction–diffusion model.

### 2.4 Gas Sensing Performance of CSMs from Atmosphere to Vacuum at RT

**FIGURE 5:**
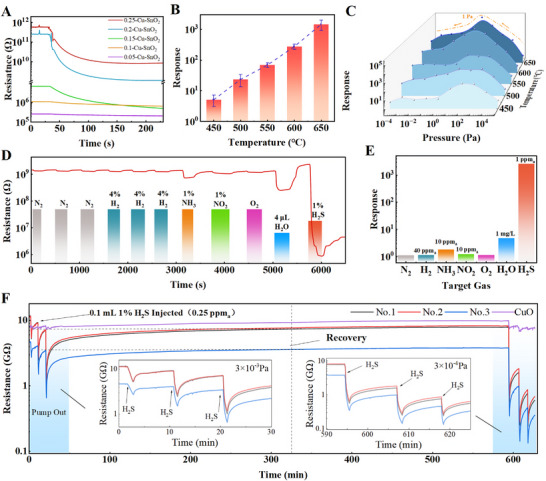
H_2_S sensing mechanism and performance of porous Cu‐doped SnO_2_ microspheres at room temperature under different vacuums. (A) Resistance–time profiles of sensors with different Cu doping levels (0.05–0.25 CSM‐550) under 3 × 10^−^^3^ Pa. (B) CSM response of varying annealing temperature under 1 ppm_a_ H_2_S at 298 K in 1 × 10^−^^3^ Pa for 200 s. Data are presented as mean ± SD (*n* = 3). (C) 1 ppm_a_ H_2_S response of CSM sensors annealed at varying temperatures under 10^5^ Pa to 10^−^^4^ Pa at 298 K.(D) Resistance of CSM‐650 under various gases (H_2_, NH_3_, NO_2_, O_2_, H_2_O, and H_2_S) at 1 × 10^−^^3^ Pa with pump cycling. (E) Response to different target gases. (F) The resistance‐time curve of 0.1 mL H_2_S injected for 50 s with long time pump. Note: ppm_a_ represents the atmospheric‐equivalent concentration, calculated as: ppm_a_ = (V_inj_/V_c_) × purity × 10^6^, see details in Section 4.3.

We investigated the gas sensing performance of the CSMs under various pressures (from 10^5^ to 10^−^^4^ Pa) at room temperature (298 K), and the obtained results are shown in Figure 5. The sensing behavior exhibits notable differences in sensing performance when the pressure drops. These response values were measured under a fixed injection of 0.4 mL 1% H_2_S, corresponding to an approximate concentration of 1 ppm_a_ within the sealed test chamber. When the pressure was reduced to 3 × 10^−^^3^ Pa, the resistance behavior of the CSMs exhibited a distinct trend depending on the Cu:Sn molar ratio. As the Cu:Sn molar ratios of CSM‐550 were tuned from 0.05 to 0.2, and the response to H_2_S increased, as shown in Figure 5A. At higher Cu loadings (≥ 0.2), the baseline resistance exceeded 100 GΩ, approaching the instrumental detection limit and introducing substantial signal noise. Although not typically optimal for terrestrial environments, a Cu:Sn molar ratio of 0.15 was selected as the optimal composition for the subsequent experiments to balance sensitivity and measurement stability under high‐vacuum conditions. When the Cu doping concentration is below 0.15, the material predominantly exhibits its *n*‐type behavior. Under decreasing vacuum pressure, surface‐adsorbed oxygen desorbs, releasing trapped electrons back into the conduction band, thereby increasing its conductivity. However, when the Cu content exceeds a certain threshold, the surface begins to exhibit p‐type semiconductor characteristics. In this regime, the reduction in adsorbed oxygen species leads to a decline in hole carrier concentration, ultimately resulting in the sensor's reduced electrical conductivity (Figure S8).

The effect of annealing temperature was investigated on the performance of 0.15‐CSM sensors under vacuum at 293 K and with exposure to 1 ppm_a_ H_2_S. As shown in Figure 5B, the response values remained at a low value for the samples annealed at 450°C (5.43) and 500°C (58.71), then increased significantly to those at 231.3 at 550°C in 200 s. A further increase in the annealing temperature up to 600°C and 650°C led to even higher responses of 321.81 and 1019.28 in 200 s, respectively.

Besides, the sensing responses of CSM sensors annealed at different temperatures (450°C–650°C) were evaluated across a wide pressure range from high vacuum (10^−^^4^ Pa) to ambient pressure (10^5^ Pa), as shown in Figure 5C. The peak at ∼1 Pa reflects a trade‐off between surface reactivity and gas supply. Above 1 Pa, vacuum enhances the response by increasing *R*_a_ and clearing active sites. Below 1 Pa, the sensor enters an oxygen supply‐limited regime (Figure 4), where insufficient molecular flux becomes the bottleneck, causing the response to drop. The results reveal a pressure‐dependent sensing performance, with a volcano‐shaped response trend centered around 10 Pa. This corresponds to the critical pressure where the molecular pump activates, causing the decrease in surface‐adsorbed oxygen and leading to the reduced sensing response under lower pressures. Notably, CSM annealed at 650°C exhibited the highest response of ∼10^5^ at 10 Pa, compared to only ∼10^2^ for the 450°C sample under the same conditions. The CSM‐650 sample still maintained a good response (∼10^3^) under 10^−^^3^ Pa, while the response of CSM‐450 sample dropped below 10. These results indicate that higher annealing temperatures promote the formation of porous architectures and active surface states, which are critical for maintaining good sensitivity under oxygen‐deficient and low‐pressure conditions. As illustrated in Figure 5D,E, the sensor showed excellent responses for H_2_S compared to other interfering gases, tested at 298 K and 10^−^^3^ Pa. When various gases were injected, e.g., 4 mL of N_2_, 4% H_2_ (in balance 40 ppm_a_), 1% NH_3_ (10 ppm_a_), and 1% NO_2_ (10 ppm_a_), the sensor's resistance displayed minimal changes, maintaining values at the 10^9^ Ω level, and gradually recovered via the pump to 10^−^^3^ Pa. In contrast, injecting 1% H_2_S (1 ppm_a_) resulted in a sharp resistance drop exceeding 10^3^, indicating a significant response, which is more than three times greater than that of O_2_ and H_2_O vapor (1 mg/L). These findings demonstrate the high sensitivity and selectivity of the porous CSM sensors toward H_2_S under room temperature and oxygen‐deficient environments, attributable to both microscopic and macroscopic structural optimizations. The high‐temperature–induced porous architecture significantly enhances gas diffusion and adsorption efficiency, enabling stronger sensing responses under vacuum conditions than in the atmosphere. Besides, we compared the response and recovery of CSM‐650 and CuO under lower H_2_S dose (0.25 ppm_a_) in Figure 5F. The CSM‐650 recovers to 80%–90% of its original resistance (baseline) after about 10 h pumping, while the resistance change of CuO is negligible.

### 2.5 H_2_S Gas Detection at Subzero and High‐Vacuum Conditions

**FIGURE 6:**
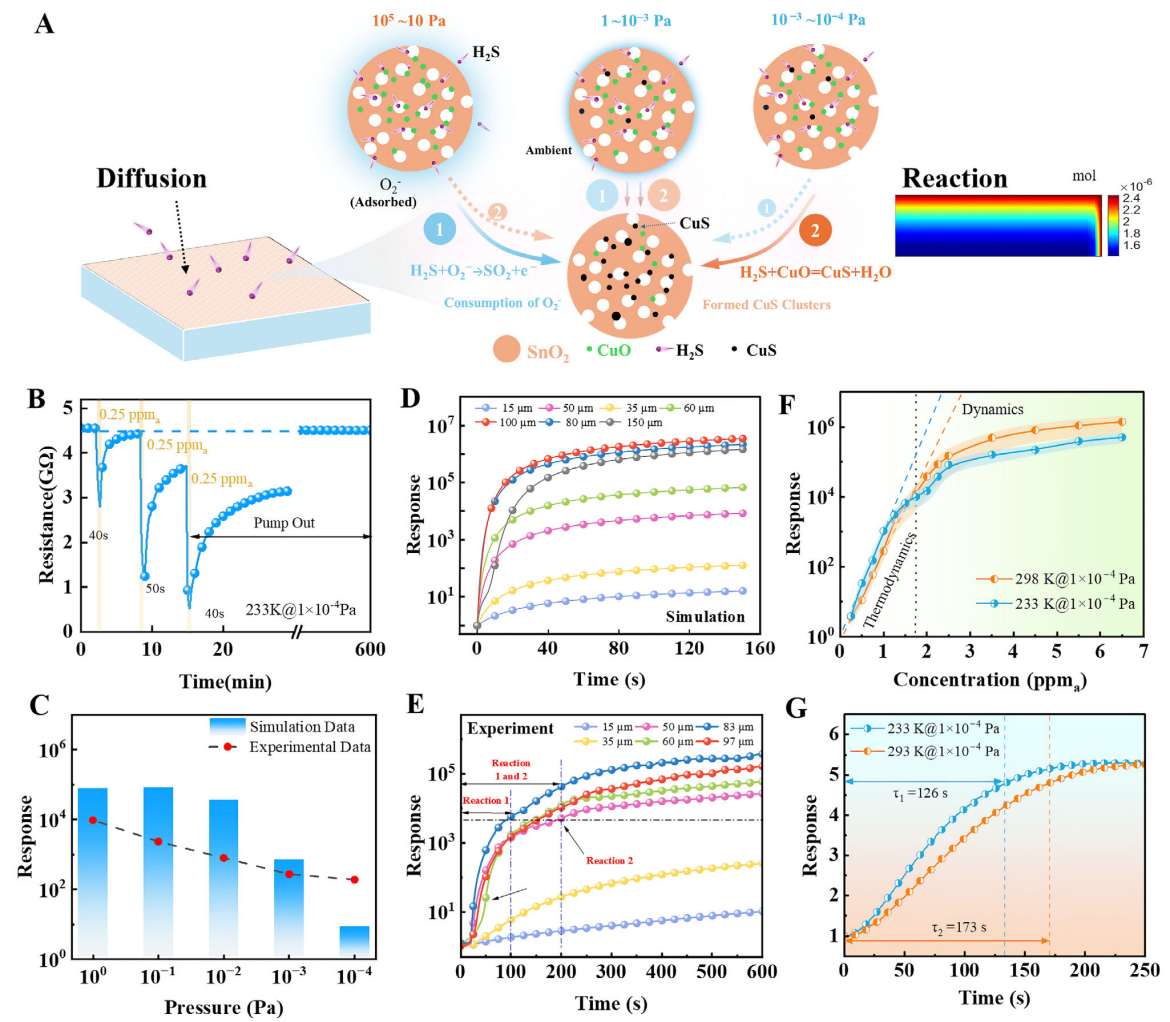
Simulation and experimental analysis of sensors (CSM‐650) under vacuum and low temperature (represented by CuS model). (A) Scheme of two main reactions. (B) The resistance‐time curve of 0.1 mL H_2_S is injected with long time pump 233 K and 10^−4^ Pa. (C) Simulation and experiment of CSM (0.15:1) for 1 ppm_a_ H_2_S under different vacuums. Time‐resolved simulated (D) and experimental response (E) under different thicknesses of CSM in 1 ppm_a_ H_2_S at 1 × 10^−3^ Pa. The response of CSM‐650 with (F) continuously injected H_2_S and (G) 0.25 ppm_a_ H_2_S under 10^−^^4^ Pa at different temperatures.

As widely reported, the sensing mechanism primarily involves two redox reactions (Figure 6A): (1) the adsorbed oxygen species (O_2_^−^) on SnO_2_ surfaces react with H_2_S; and (2) the in situ chemical reaction with H_2_S. In our theoretical framework, CuS serves as a functional reference state to represent the Cu_x_S, facilitating the self‐consistent modeling of the energy band alignment at the interface. Besides, the carrier density and oxygen adsorption behavior of SMOs are both remarkably altered under vacuum conditions (Figure 2 and Figures S4–S8). Finite element analysis (FEA) and experimental investigations were further carried out to elucidate CSM sensors' performance under different pressures at 300 K. The packed‐bed reactor module in COMOSL Multiphysics was used to analyze the physical and chemical processes occurring in the sensor during the detection process (Figure 6A). As shown in the simulation results, under the conditions of 10^−^^3^ Pa and 300 K, the H_2_S molecules gradually diffused into the CSM porous structures from the right and upper boundaries. The fundamental details of the coupled model with mass transfer and responses are provided in Figure S9 and Table S3. The model is semi‐quantitative and is intended to capture the dominant environmental trends, rather than to provide a fully atomistic description. Microscopic depletion‐layer variations within individual grains are not explicitly resolved, which is an engineering‐scale approximation for porous SMO films with low Cu doping. When the equilibrium was reached, the 50 µm‐thick sensing film became fully permeated by 1 ppm_a_ H_2_S.

Surface concentration of the resulting CuS exhibited a gradient change, which gradually decreased from the outer surface toward the interior. The sensing process was continuous until the chemical reaction reached the completion stage. Figure 6B shows almost full recovery of CSM‐650 under 10^−^^4^ Pa at 233 K, which is superior to its behavior under ∼10^−^^3^ Pa at 293 K. This enhanced recovery arises mainly because the lower pressure and temperature suppress irreversible surface reactions and favor desorption of weakly bound species. Therefore, partial recovery reflects the thermodynamic–kinetic constraints of the extreme environment rather than material degradation. After prolonged evacuation in Figures 5F and 6B, the baseline approaches a stable quasi‐steady state, indicating operational stability within the intended window.

As the pressure decreased from 1 to 10^−^^4^ Pa, the response value gradually decreased from 10^6^ to approximately 100, as shown in Figure 6C. This reduction reflects the semi‐quantitative nature of our model, which accounts only for oxygen‐related band bending and does not include the extra barrier introduced by CuS formation, leading to an underestimation in deep vacuum. At a moderate vacuum level of 10^−^^3^ Pa, the sensor exhibited nearly complete recovery at room temperature upon exposure to 1 ppm_a_ H_2_S for three cycles in Figure S10 at 10^−^^3^ Pa. However, the responses gradually diminished as the temperature was further decreased. Under a pressure of 10^−^^4^ Pa at 298 K, the sensing performance significantly deteriorated, with the response decreasing from 965.31 to 330.8 after just one cycle. Figure S10 indicates that the response failed to recover at 1 ppm_a_ after three successive tests under the condition of 233 K and 10^−^^4^ Pa, suggesting that H_2_S desorption was severely hindered. The distinctive sensing behavior highlights the unique characteristics of the gas–solid reaction mechanism of porous CSM under vacuum conditions. The kinetics of the reaction were influenced by the concentration of O_2_^−^ and the chemical reactivity of H_2_S at 300 K.

We further investigated the effect of film thickness on sensing performance to elucidate the governing mechanisms under such conditions. The correlation between film thickness and sensor responses under different pressures offers insights into the cooperative reaction mechanisms governing the sensing process. Figure 6D presents simulated temporal concentration evolutions for the CSM‐650 sensing layer with varying thicknesses from 15–150 µm to further elucidate this behavior. The responses were found to increase with thickness below 100 µm, but it decreased when the thickness was over 150 µm. H_2_S molecules cannot penetrate deep enough within the measurement time window, leading to incomplete utilization of the film volume. This behavior is well aligned with the experimental results (Figure 6E), confirming the critical influence of film thickness on sensing efficiency. Specifically, film thickness directly modulates the mass transport of H_2_S molecules through the hierarchical porous architecture, thereby determining the accessibility of the target gas to the buried reactive sites. The initial resistance drop is attributed to the consumption of residual surface‐adsorbed O_2_^−^ by H_2_S, which reacts rapidly with the incoming H_2_S, releasing electrons into the conduction band (Reaction 1). As the H_2_S penetrates further into the film, it undergoes a secondary chemical reaction (represented by the CuS model) forming sulfurized species and inducing additional charge transfer (Reaction 2). The total resistance change reflects the combined effects of surface redox interactions and bulk‐phase gas–solid reactions. This simulation trend is well‐supported by experimental data (Figure 6E), which exhibits consistent thickness‐dependent response patterns. Collectively, these results reveal a two‐stage sensing mechanism, involving an initial fast surface‐limited reaction followed by a slower bulk‐phase process, controlled by gas diffusion and reaction depth. This dual‐pathway understanding is essential for the rational design of sensor architectures to optimiz their performance under vacuum and other extreme environments.

Gas sensing tests were further conducted under −40°C and 10^−^^4^ Pa to simulate key features of space environments, enabling assessment of sensor performance under extreme conditions. Reusability degradation behaviors under extreme conditions in Figure 6F and Figure S11 reveal the underlying sensing mechanism by continuously injected. As the gas exposure progresses, the slower surface reaction kinetics at cryogenic temperatures, which stem from reduced molecular thermal energy and weakened adsorption interactions, gradually dominate, leading to delayed saturation and lower responses at 298 K. The response value (above 1.5 ppm_a_) of H_2_S at 233 K was less than that at 298 K, indicating that the sensing process was predominantly governed by the chemical reactions following the adsorption saturation. This phenomenon is mainly attributed to reduced molecular kinetics and lower adsorption energy at subzero temperatures, which influence both gas diffusion and surface reaction rates. These combined effects lead to an initial sharp drop of resistance, indicating a kinetically favorable reaction pathway in the early stages of exposure.

The dynamic responses toward 0.25 ppm_a_ H_2_S at different temperatures were studied, and the results (Figure 6G) reveal that lower temperatures resulted in faster response kinetics (*τ*_1_ = 126 s vs. *τ*_2_ = 173 s). This apparent contradiction arises from two competing mechanisms. (i) The vacuum environment enhances molecular transport efficiency due to the extended mean free path and diminished gas‐gas collisions, accelerating H_2_S delivery to active sensing sites while suppressing desorption; (ii) At trace H_2_S concentrations, unsaturated surface‐active sites (e.g., oxygen vacancies, CuO domains) enable rapid adsorption and immediate charge transfer upon molecular arrival, triggering an initial sharp resistance decrease indicative of kinetically favorable early‐stage reactions. This transition from a kinetically dominated regime (moderate vacuum) to a thermodynamically governed regime (ultrahigh vacuum and cryogenic temperature) underlines the good sensitivity of SMO‐based sensors to environmental extremes.

Under cryogenic and high‐vacuum conditions, CSM‐650 shows near‐complete recovery at low H_2_S doses but only partial recovery at higher doses. This coverage‐dependent behavior indicates that mild exposure is largely reversible, whereas excessive H_2_S drives deeper sulfidation of Cu‐related sites that cannot be readily re‐oxidized in oxygen‐defect's environments. Accordingly, reliable sensing for extraterrestrial applications requires balancing adsorption strength with active‐site regeneration and desorption kinetics, rather than merely maximizing sensitivity. Our approach combines the following merits, i.e., (i) hierarchical porosity to facilitate gas diffusion and adsorption on the surface (e.g., Fickian diffusion in air vs. ballistic transport under vacuum) [36]; (ii) doping and oxygen vacancy modulation for enhanced conductivity and chemisorption for both atmosphere and oxygen defects environment; (iii) heterojunction interfaces to sustain gas‐induced charge transport. Besides, the Cu–S interaction should be viewed as a privileged reaction pathway within a more general surface–bulk–interface framework for selectivity, rather than as a restriction that limits the sensor only to sulfur‐containing gases. Building on this principle, our oxygen‐independent framework decouples sensor functionality from ambient O_2_ and sustains performance across wide‐ranging conditions—from atmospheric pressure to high vacuum and from room temperature to cryogenic regimes.

## 3 Discussion

We have developed a straightforward strategy to fabricate porous SnO_2_ microspheres that contain abundant oxygen vacancies and allow for tunable Cu doping. The coupling of different levels of engineering methods of “surface‐bulk‐interface” provides a unique platform for H_2_S gas sensing applications in both ambient conditions on Earth and simulated extraterrestrial environments. The sensor maintains functionality across a wide pressure gradient from 10^5^ to 10^−4^ Pa and temperatures down to −40°C, exhibiting an environment‐responsive transition from surface oxygen‐mediated to interface‐driven pathways. As a foundational probe, this study establishes a vital physical baseline by defining the 10^−4^ Pa mechanistic threshold and the supply‐limited kinetic regime. While actual deep‐space deployment faces greater complexities, such as ionizing radiation and more extreme gradients, these findings provide a strategic roadmap for future space‐hardened systems, where research will prioritize the coupling effects of high‐energy particles on lattice defect dynamics. Our method acts as a technological bridge, facilitating the translation of advanced material innovations into scalable platforms for extraterrestrial sensing systems and terrestrial applications, paving the way for gas detection by SMOs in future space exploration.

## 4 Materials and Methods

### 4.1 Fabrication of Cu‐Doped Porous SnO_2_ Microspheres

*Cu‐doped SnO_2_ microspheres* were synthesized via a solvothermal method. First, 2 mmol of SnCl_2_·2H_2_O was dissolved in the mixture to form a clear solution (denoted as Solution A), which was heated at 75°C in a water bath for 2 h and then left to stand overnight. Subsequently, Solution A was transferred into a 50 mL Teflon‐lined autoclave, and 3 mL of ethylene glycol (EG) was added. The mixture was stirred for 30 min. CuCl_2_·2H_2_O was added before a solvothermal treatment was performed at 180°C for 12 h. The resulting products were centrifuged, washed with anhydrous ethanol, and dried at 60°C for 12 h, followed by annealing at 550°C for 1 hour with a heating rate of 5°C/min. Products with Cu: Sn molar ratios n:1 (*n* = 0.05, 0.1, 0.15, 0.2, 0.25) were denoted as n‐CSM, while the undoped products were labeled SM.

### 4.2 Characterization of Microspheres

Morphology of the sensing materials was characterized using field effect‐SEM (FESEM, Gemini SEM 300, Carl Zeiss) and TEM (JEM‐2100, JEOL). Elemental distributions were analyzed via energy‐dispersive X‐ray spectroscopy (EDS) mapping using the Gemini SEM 300 system. XRD measurements were performed using an XRD‐7000 diffractometer (Shimadzu Corporation) within a 2θ range of 20° to 80°. X‐ray photoelectron spectroscopy (XPS) spectra were acquired using an AXIS Supra+ system (Kratos), and Raman spectra were recorded using a Raman microscope (DXR 2xi, Thermo Fisher Scientific). The VK‐X3000 laser microscope from Keyence was used to measure the thickness of the film (Figure S12).

### 4.3 Gas Sensing Experiments

The responses of the SnO_2_ gas sensor were expressed as *R*_a_/*R*_g_, where *R*_a_ and *R*_g_ are the resistance of SnO_2_ microstructures exposed to the air (or vacuum) and the target gaseous environment, respectively. The resistances of the devices were measured by a commercial gas sensor system (JF02F, Guiyanjinfeng, China), within a range of 1 Ω–100 GΩ. The gas sensor was measured in a low‐temperature and high‐vacuum test system, as shown in Figure S1. The testing system was comprised of four components, i.e., a vacuum equipment set, a temperature‐controlled testing chamber with a cold trap, a custom‐made inlet unit, and a system for acquiring gas sensor resistance measurements. The system could lower the temperature inside the testing chamber down to −40°C, achieving a vacuum level of 1×10^−4^ Pa. Due to the uniqueness of the vacuum environment, the conventional “parts per million (ppm)” concentration defined under atmospheric pressure cannot be directly applied. In this study, ppm_a_ (parts per million at atmospheric equivalence, *C*_eq_) is defined as the equivalent concentration corresponding to the volumetric ratio (*y*_0_) of the injected target gas (*V*_inj_) to the total chamber volume (*V*_c_) under standard atmospheric conditions (10^5^ Pa), as the total number of injected molecules is independent of the vacuum level.

$$C_{\mathrm{eq}}=f\left(\frac{V_{\mathrm{inj}}}{V_{c}}\right)=y_{0}\frac{V_{\mathrm{inj}}}{V_{c}}\times 10^{6}$$

For instance, 4 mL of H_2_S (1%, N_2_ rest) was injected using a microliter syringe into a 4 L pre‐evacuated chamber, resulting in a final concentration of 10 ppm_a_ under vacuum. This parameter enables a unified representation of gas concentration across pressure regimes, facilitating direct comparison between measurements performed under atmospheric and high‐vacuum environments. The gas delivery was conducted through a three‐chamber system consisting of a reservoir chamber A (∼5 mL), a buffer chamber B (∼20 mL), and a main detection chamber C (4 L), as shown in Figure S1. This configuration allows precise control of gas dosage, stabilization of pressure gradients, and full equilibration before measurement.

### 4.4 Computational Methods and Numerical Simulations

The theoretical calculations were carried out for the material in the framework of Density Functional Theory (DFT) using the Vienna Ab initio Simulation Package (VASP 6.3.0). The generalized gradient approximation (GGA) of the Perdew‐Burke‐Ernzerhof (PBE) functional was used to describe the exchange‐correlation energy. The projected augmented wave (PAW) method and pseudopotentials were used to describe the interactions between valence electrons and ions. To ensure the efficiency of the computational results and parallel computing, a 2 × 3 × 1 *k*‐point grid under Monkhorst‐Pack was used in the optimization process and 450 eV truncation energy was set. To correct for the localization effect of d‐orbital electrons in transition metal atoms, the Hubbard U plus method was used, with effective U values (Ueff) of 4.7 eV for both Sn and Cu. The lattice parameters and ionic positions of all crystals were fully relaxed, and the convergence criteria for the total energy of all relaxed atoms and the final force were 10^−5^ eV and 0.03 eV/Å, respectively. In addition, electrical and thermal transport properties were analyzed using semi‐empirical Boltzmann transport theory. These simulations provided insight into temperature‐ and defect‐dependent conductivity of the materials. The numerical simulations, including pressure‐dependent resistance and surface potential evolution, were performed using MATLAB R2024b, based on Wolkenstein adsorption theory. The finite element method (FEM) simulations were performed using COMSOL Multiphysics 5.5.

### 4.5 Statistical Analysis

To ensure reproducibility and minimize device‐history effects, concentration‐dependent and comparative gas sensing measurements were conducted using fresh sensors, except for low‐dose cycling and cumulative‐dose protocols where reuse of the same device was required to track baseline evolution. For the core sensing performance evaluations across the atmosphere‐to‐vacuum gradient (Figures 5 and 6), sensors fabricated with four dripping cycles (D4) were used as a standard configuration. Gas sensing responses were evaluated based on deterministic resistive parameters obtained under controlled exposure conditions. Repeated gas on/off cycles were employed to assess operational repeatability, and representative response values were extracted from steady‐state regions. Where applicable, data are presented as mean values with standard deviations (SD) reflecting device‐level fluctuations; selected characterization results are shown as representative measurements. No inferential statistical tests (e.g., *t*‐tests or ANOVA) were applied, as the reported metrics represent defined physical quantities rather than stochastic outcomes of group‐wise comparisons. Data processing and visualization were performed using Origin 2025 and MATLAB.

## Author Contributions

Conceptualization: WL, XC; Methodology and Simulation: XC, JXC; Investigation and Experiment: XC, KXC, TSY, JHC, YLY; Visualization: XC, YHL, YY; Supervision: WL, YQF; Writing – original draft: XC, JXC; Writing – review & editing: YQF, WL.

## Funding

Science Challenge Project (No.TZ2025011).

## Conflicts of Interest

The authors declare no conflicts of interest.

## Supporting information




**Supporting File**: advs74103‐sup‐0001‐SuppMat.docx.

## Data Availability

The data that support the findings of this study are available in the supplementary material of this article.
