# Supplementary material for: Wide‐Range Adaptive Metal Oxide for Hydrogen Sulfide Detection From Earth to Space‐Like Environments
Source: Adv Sci (Weinh). 2026 Feb 8;13(19):e15684. doi: 10.1002/advs.202515684 (PMC13045391; doi:10.1002/advs.202515684)
Supplement: Supplementary file 1 — Supporting File: advs74103‐sup‐0001‐SuppMat.docx. [file ADVS-13-e15684-s001.docx]

Supporting Information

**Wide**-**Range Adaptive Metal Oxide for Hydrogen Sulfide Detection from Earth to Space-like Environments**

*Xi Chen, Jiaxin Chen, Yanghui Liu, Junhao Cao, Yang Yuan, Kaixin Chen, Yilang Ye, Tongshuai Yang, Yongqing Fu* and Wei Luo **

We first calculated how oxygen adsorption affects the surface potential

Vs*Vs*

using the Wolkenstein/Langmuir model. Then, the obtained

Vs(P)*Vs(P)*

and conductivity trend were imported into COMSOL to simulate the macroscopic competition between oxygen desorption and the CuO–H_2_S reaction in the porous sensing layer.

Phase I — Theoretical Modeling of Oxygen Adsorption (MATLAB)

Electrical conductivity depends on the relationship between the concentration of adsorbed oxygen and the surface charge of the semiconductor. Surface coverage refers to the extent to which the semiconductor surface is covered by adsorbed molecules, defined as the ratio of the number of adsorbed molecules to the number of available surface states per unit area.

Based on Langmuir's monolayer adsorption theory, surface coverage (θ) can be expressed quantitatively as a function of pressure at adsorption-desorption equilibrium:

θ=NN∗=βPβP+1#(1.2)*𝜃=NN∗=𝛽P𝛽P+1#1.2*

Using the Wolkenstein adsorption model(S1), which assumes that the total surface charge is caused solely by adsorbed molecules, the total surface charge can be written as the difference between adsorption (a(N)) and desorption (d(N)) rates.

N∗*N∗*

is the number of adsorption sites per unit area,

N0*N0*

and

N−*N−*

are the numbers of neutral adsorbates and chemisorbed ions per unit area, respectively, with

N = N0 + N−*N = N0 + N−*

representing the total number of adsorbed particles per unit aread. q^0^ is the binding potential for neutral chemisorption states.

dNdt=a(N)−d(N)= αp(N∗−N)−(N∗θ−ϑ−exp(−q0+(Ebc−Ess)kT)+N∗θ0ϑ0exp(−q0kT))#(1.3)*dNdt=aN−dN= 𝛼pN∗−N−N∗𝜃−𝜗−exp⁡−q0+Ecb−EsskT+N∗𝜃0𝜗0exp⁡−q0kT#1.3*

Because adsorption depends on pressure, a control factor α is defined by the following equation(*S2*):

α=s0∙∂2πMkT√#(1.4)*𝛼=s0∙𝜕2𝜋MkT#1.4*

Here,

s0*s0*

represents the binding potential of neutral chemisorption states,

∂*𝜕*

is the effective surface area of adsorbed molecules, and

M*M*

is the molecular mass of the adsorbed molecule (oxygen O₂). This expression describes the adsorption rate at a specific temperature (T) for molecules with mass M and effective surface area, reflecting the overall probability and efficiency of the adsorption reaction.

Ebc*Ecb*

is the energy level of conduction band electrons in SnO₂, while

Ess *Ess*

is the energy level of chemisorption surface states.

ϑ0*𝜗0*

and

ϑ−*𝜗−*

are the vibration frequencies of the corresponding molecules, typically around 10^13^ Hz(*S1*).

Non-dissociative chemisorption refers to the process where gas molecules are adsorbed onto the surface without breaking molecular bonds, retaining their original structure. In contrast, dissociative chemisorption involves the breaking of molecular bonds, where molecules dissociate into atoms or simpler molecules during adsorption, forming stronger chemical bonds. In dissociative adsorption, colliding molecules dissociate into two free radicals, and a second anion must find a nearby vacancy to adsorb onto(*S3*).

For n-type and p-type semiconductors during non-dissociative adsorption, the oxygen species (initially adsorbed in a neutral state) transition from surface states (adsorption sites) to conduction bands or other states by overcoming potential barriers:

s0⋅∂2πMkT√(1−θ)=N∗θ−ϑ−exp(−q0+(Ebc−Ess)kT)+N∗θ0ϑ0exp(−q0kT)#(1.5)*s0⋅∂2𝜋MkT1−𝜃=N∗𝜃−𝜗−exp⁡−q0+Ecb−EsskT+N∗𝜃0𝜗0exp⁡−q0kT#1.5*

Considering the orbital spin of oxygen, the degeneracy factor g = 2. For both n-type and p-type semiconductors, the equation simplifies:

βn=β0[1+12exp(EF−EsskT)]×[1+ϑ−2ϑ0exp(−Ebc−EFkT)]−1#(1.6)*𝛽n=𝛽01+12exp⁡EF−EsskT×1+𝜗−2𝜗0exp⁡−Ecb−EFkT−1#1.6*

Where

β0=s0N∗ν02πMkT√exp(q0kT)#(1.7)*𝛽0=s0N∗𝜈02𝜋MkTexp⁡q0kT#1.7*

Due to adsorption equilibrium, the surface charge density can be derived under the charge neutrality condition:

Qs+Qsc=0#(1.8)*Qs+Qsc=0#1.8*

Here,

Qs*Qs*

is the surface charge density, and

Qsc*Qsc*

is the charge density in the semiconductor's space charge region. Adsorption equilibrium is expressed as(*S4*):

Qs= −eN−= −efFDθN∗#(1.9)*Qs= −eN−= −efFD𝜃N∗#1.9*

The Fermi-Dirac distribution function

fFD  *fFD*

can be written as:

fFD=[1+12exp(−Ef−EsskT)]−1=[1+12exp(−Esc−EsskT)exp(Ebc−EFkT)×exp(eVskT)]−1#(1.10)*fFD=1+12exp⁡−Ef−EsskT−1=1+12exp⁡−Ecs−EsskTexp⁡Ecb−EFkT×exp⁡eVskT−1#1.10*

Further, the space charge density

Qsc*Qsc*

can be calculated using Poisson's equation and Gauss's theorem(*S4*), with surface potential

Vs*Vs*

as a function:

Qsc= 2–√(nb+pb)eLD[cosh(ub+eVskT)coshub−eVskTtanhub−1]−−−−−−−−−−−−−−−−−−−−−−−−−⎷#(1.11)*Qsc= 2nb+pbeLDcosh⁡ub+eVskTcosh⁡ub−eVskTtanh⁡ub−1#1.11*

Another expression is:

Qsc(ψs)= 2–√(nb+pb)eLDeub(eψs−ψs−1)+e−ub(e−ψs+ψs+1)eub+e−ub −−−−−−−−−−−−−−−−−−−−√ #(1.12)*Qsc𝜓s= 2nb+pbeLDeube𝜓s−𝜓s−1+e−ube−𝜓s+𝜓s+1eub+e−ub #1.12*

Where

nb*nb*

and

pb*pb*

are the concentrations of free electrons and holes inside the semiconductor, with representing electron mobility

ub=EF−EikT=ln(nbni)*ub=EF−EikT=ln⁡(nbni)*

.

ni*ni*

is the intrinsic carrier concentration of semiconductor material. The Debye length

LD*LD*

is expressed simply as:

LD= εε0kTnbq2−−−−√#(1.13)*LD= 𝜀𝜀0kTnbq2#1.13*

The expression for

ψs*𝜓s*

is:

ψs=eVskT#(1.14)*𝜓s=eVskT#1.14*

According to the charge neutrality condition:

Qs(Vs)T,p+Qsc(Vs)T=0#(1.15)*QsVsT,p+QscVsT=0#1.15*

By solving Eq1.14, the equilibrium surface potential

Vs*Vs*

is obtained. The total surface coverage

θ*𝜃*

can be calculated by Eq1.2. After obtaining the total surface coverage value, the coverage of negative charge states and neutral adsorbates can be computed using the following relationship:

θ−=fFDθ θ0=1−fFDθ#(1.16)*𝜃−=fFD𝜃 𝜃0=1−fFD𝜃#1.16*

The numerical calculations in the presented work have been performed by a computer programmed written in R2022b version of MATLAB software. The parameters used in the numerical calculations have been shown in Table S2.

Phase II — Coupled Gas–Solid Interaction Semi-Quantitative Simulation (COMSOL)

According to relevant literature and the built-in physics modules of COMSOL Multiphysics, the *Chemical Reaction Engineering* module is suitable for simulating surface oxygen adsorption dynamics and subsequent chemical reactions. Given the nanoscale hierarchical porous microspheres with 3D interconnected channels on the sensor surface, explicitly modeling each adsorption and reaction event is computationally impractical. Instead, the *Packed Bed Reactor* module employs a homogenization approach that treats the porous structure as a uniform medium of catalyst and fluid phases. Mass transport is characterized by parameters such as porosity and effective diffusivity. The sensor's exposure to target gases is modeled by coupling the gas chamber domain (as shown in Figure S1) with the porous medium domain based on the homogenized model.( Considering the correlation between adsorbed oxygen concentration and the resistance of SnO_2_, a linear approximation is adopted in this preliminary model.) The total resistance is calculated by combining the amount of reaction products with the conductivity module. The reaction leads to the formation of highly conductive CuS on the sensing film, reducing the overall resistance between interdigitated electrodes. The following mixing rule describes the effective conductivity:

The sensor’s exposure to target gases is modeled by coupling the homogenized porous domain with the gas chamber domain (as shown in Figure S1). This COMSOL simulation framework incorporates key outputs from prior MATLAB simulations (Figure S5–S7), where the distribution of oxygen adsorption sites (denoted as G_0_) was determined by the surface coverage θ of adsorbed species on the semiconductor surface. In particular, the adsorbed oxygen concentration is directly linked to the surface potential Vs, which modulates the local carrier density and hence the conductivity of the SnO₂ layer. To capture this effect, a linearized approximation is introduced in COMSOL to relate the adsorbed oxygen concentration to the effective conductivity of SnO₂.

In addition to oxygen adsorption, the simulation also considers the reaction between adsorbed H_2_S and the CuO component, which forms highly conductive CuS (represented by a metal-suflide cluster model) on the sensing film and reduces the overall resistance between interdigitated electrodes. The total resistance is calculated by combining the amount of reaction products with the conductivity module, and the effective conductivity of the sensing layer is described by the following mixing rule:


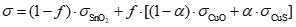


Where *f* is the Cu content fraction, and *α* represents the conversion ratio from CuO to CuS.

**Table S1. The surface area of CSM under different annealing temperatures**

| Material | Monolayer adsorption volume（cm^3^/g） | Surface area（m^2^/g） | Adsorption constant C | Average pore size（nm） |
| --- | --- | --- | --- | --- |
| CSM-450 | 13.625 | 59.302 | 75.636 | 8.1581 |
| CSM-550 | 7.5397 | 32.816 | 130.86 | 13.997 |
| CSM-650 | 4.1247 | 17.953 | 260.99 | 19.498 |

**Table S2. The parameter of semiconductors**

| Physical Property | Symbol | N_type | P_type |
| --- | --- | --- | --- |
| Band gap | Eg*Eg* | 3.6 eV(*S5*) | 1.6 eV(*S6*) |
| Energy level of the induced states caused by chemical adsorption | ESC−Ess*ECS−Ess* | 1 eV(*S9*) | 2.5 eV |
| Degeneracy factor | ga*ga* | 2 | 2 |
| Effective mass for electrons in conduction band | m∗e*me∗* | 0.3 m_0_(*S5*) | 0.3 m_0_ |
| Effective mass for holes in valence band | m∗h*mh∗* | 0.3 m_0_(*S5*) | 0.3 m_0_ |
| Free Electron mass | m0*m0* | 0.91094 × 10^–30^  kg | 0.91094 × 10^–30^ kg |
| Dielectric constant of metal oxide semiconductor | ε*𝜀* | 12(*S5*) | 17(*S7*) |
| Density of the available surface states | N∗*N∗* | 10^19^ m^-2^ | 10^19^ m^-2^ |
| Condensation coefficient | s0*s0* | 0.1(*S3*) | 0.01(*S3*) |
| Binding of neutral chemisorbed state | q0*q0* | 0.1 eV(*S10*) | 0.2 eV(*S8*) |
| Oscillation frequency of the neutral chemisorbed state | v0*v0* | 10^13^ Hz(*S1*) | 10^13^ Hz(*S1*) |
| Oscillation frequency of the charged chemisorbed state | v−*v−* | 10^13^ Hz(*S1*) | 10^13^ Hz(*S1*) |
| Molecular mass of O_2_ | M*M* | 32 a.m.u | 32 a.m.u |

**Table S3. Simulation parameters of CSM gas sensors**

| **Parameter** | **Value** | **Unit** | **Description** |
| --- | --- | --- | --- |
| σCuS*𝜎CuS* | 0.1(*S11*) | [S/cm] | Electrical conductivity of CuS |
| σCuO*𝜎CuO* | 0.001(*S12*) | [S/cm] | Electrical conductivity of CuO |
| f*f* | 0.15 | 1 | Loading ratio of CuO |
| w*w* | 3 | [mm] | Film length |
| l*l* | 2.79 | [mm] | Film width |
| h0*h0* | 50 | [  μ*μ*  m] | Film thickness |
| r*r* | 0.5 | [  μ*μ*  m] | Particle radius in the reaction bed |
| q*q* | 0.3 | 1 | Porosity |
| CH2S*CH2S* | 1 | ppm_a_ | Inlet concentration of H_2_S |

Table S4. The summary of Vs under different N_D_

| Ref. | p(O_2_) Range (atm) | T Range (K) | Reported Vₛ (eV) | N_D_ (cm⁻³) |
| --- | --- | --- | --- | --- |
| S13 | 10⁻^10^–1 atm | 400 | 0–0.85 | 10^12^–10^18^ |
| S14 | 10⁻^13^–1 atm | 300\400\500 | 0–0.48 | 10^16^ |
| S15 | 10⁻^5^–10⁻^1^ atm* | 600 | 0–0.7 | 10^18^ |
| S16 | 10⁻^12^–1 atm | 300 – 600 | 0–0.6 | 10^18^ |
| S17 | 10⁻^6^–10⁻^1^ atm | 480 | 0~0.8 | 10^18^ |
| **This work** | **10⁻^6^–10⁻^1^ atm** | **200 – 300** | **0–0.5** | **10^15^–10¹⁸** |

*Calculated by surface state density of negatively charged oxygen

**References**

1. T. Wolkenstein, Electronic Processes on Semiconductor Surfaces during Chemisorption, Consultants, New York, 1991, pp. 35–182.
2. Kumar, A.; Kumar, M.; Kumar, R.; Singh, R.; Prasad, B.; Kumar, D. Numerical Modelling of Chemisorption of Oxygen Gas Molecules on the Surface of Semiconductor for Gas Sensors Applications. *Materials Today: Proceedings* **2019**, *18*, 1272–1279.
3. H. Lüth, Surfaces and Interfaces of Solid Materials, 3rd ed., (2001).
4. M. Winfried, Semiconductor Surfaces and Interfaces, 3rd ed., (2001), pp. 13–65.
5. V. Lantto, Semiconductor gas sensors based on SnO_2_ thick films, in: G. Sberveglieri (Ed.), Gas Sensors, Kluwer Academic Publishers, Dordrecht, 1992, pp. 117–167.
6. Korell, Lukas, et al. On the structural evolution of nanoporous optically transparent CuO photocathodes upon calcination for photoelectrochemical applications. *Nanoscale Advances* 6.11 (2024): 2875-2891.
7. Messaoud, Ouarda Ben, et al. Optoelectronic and dielectric properties of tenorite CuO thin films sprayed at various molar concentrations. *Periodica Polytechnica Chemical Engineering* 68.1 (2024): 93-105.
8. Sun, Shujuan, et al. Density functional theory study of the adsorption and dissociation of O_2_ on CuO (1 1 1) surface. *Applied Surface Science* 333 (2015): 229-234.
9. V. Lantto, P.R. Romppainen, Electrical studies on the reactions of CO with different oxygen species on SnO_2_ surfaces, Surf. Sci. 192 (1987) 243–264.
10. Rothschild, A.; Komem, Y. Numerical Computation of Chemisorption Isotherms for Device Modeling of Semiconductor Gas Sensors. 2003.
11. Hiramatsu, Hidenori, et al. Electrical conductivity control in transparent p-type (LaO) CuS thin films prepared by rf sputtering. Journal of applied physics 91.11 (2002): 9177-9181.
12. Raj, AS Aiswarya, and V. Biju. Nanostructured CuO: Facile synthesis, optical absorption and defect dependent electrical conductivity. Materials Science in Semiconductor Processing 68 (2017): 38-47.
13. Rothschild, A.; Komem, Y. Numerical Computation of Chemisorption Isotherms for Device Modeling of Semiconductor Gas Sensors. Sensors and Actuators B 93 (2003): 362–369.
14. Kumar, A.; et al. Electrical and Structural Properties of SnO₂-Based Metal Oxide Sensors: A Materials Today Proceedings Study. Materials Today: Proceedings 18 (2019): 1272–1279.
15. Kumar, A.; et al. Effect of Surface State Density on Chemisorption, Grain Potential and Carrier Concentration for SnO₂ Nanostructures. Arabian Journal for Science and Engineering 45 (2020): (2020). doi:10.1007/s13369-020-04630-3.
16. Kumar, M.; et al. Influence of Oxygen Chemisorption on Electrical Transport in SnO₂ Nanostructures. Materials Science in Semiconductor Processing 90 (2019): 236–244.
17. Gupta, R.; et al. Numerical Modeling of Chemisorption and Grain-Boundary Potential Barriers in Metal Oxide Gas Sensors. Micro & Nanostructures 171 (2022): 207423.

| 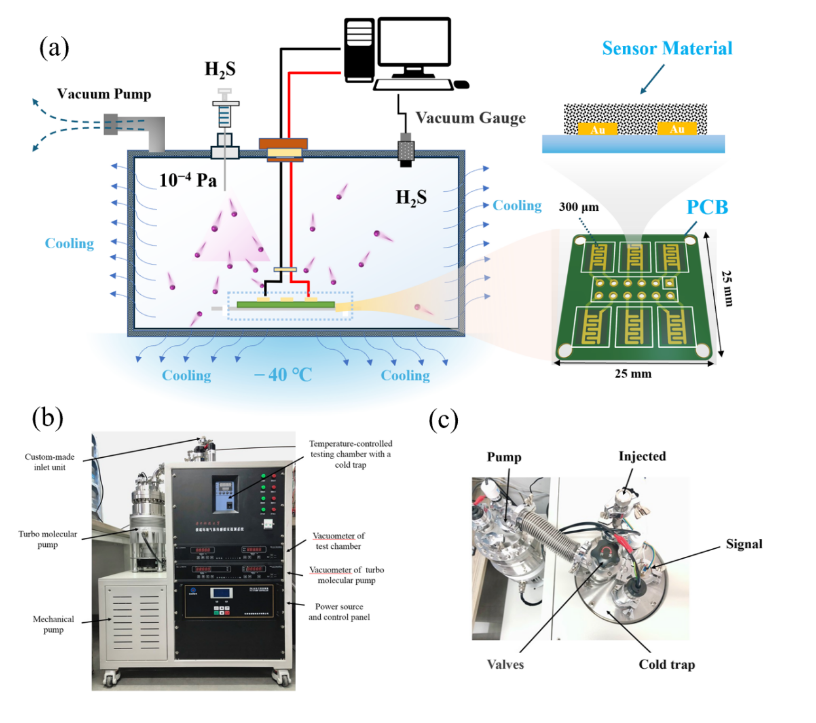 | |
| --- | --- |
| (d) | 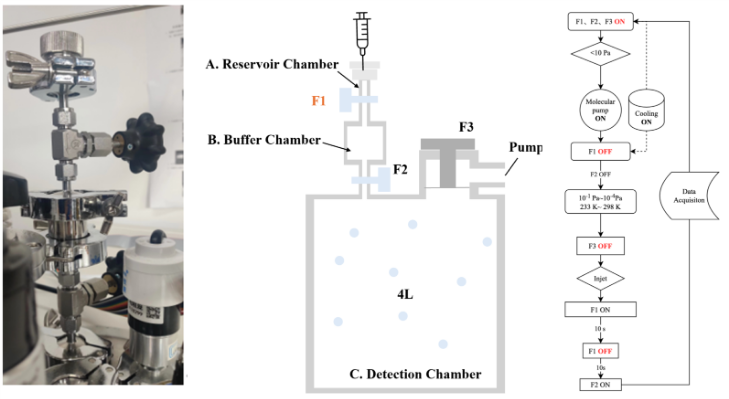 |

**Figure S1. Gas sensor test system under low temperature and high vacuum.** (a) The scheme of simulated space environments. (b) Front view and (c) Side view of the system. (d) The workflow of our experiment.


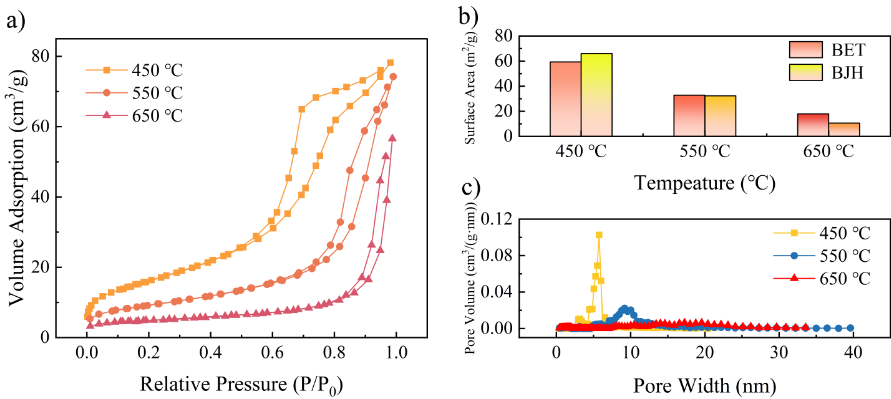


**Figure S2. The surface area and pore size distribution of the CSM samples calcined at different temperatures.** (a) N_2_ adsorption–desorption isotherms. (b) BET(BJH) specific surface areas of the samples calcined at three different temperatures. (c) Pore size distributions correspond to each calcination temperature.


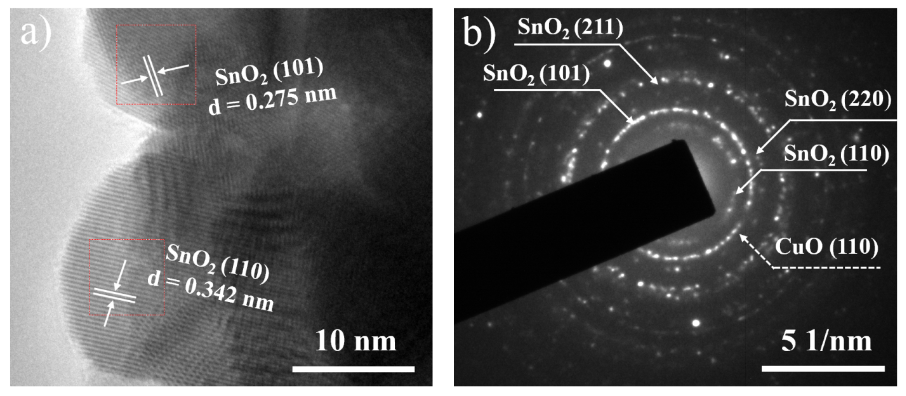


**Figure S3.TEM image of CSM-650 with a Cu/Sn molar ratio of 0.15.** (a) High-resolution TEM image of CSM-650 with a Cu/Sn molar ratio of 0.15, showing clear lattice fringes corresponding to SnO_2_ (101) and (110) planes with interplanar spacings of 0.275 nm and 0.342 nm, respectively. (b) Corresponding selected area electron diffraction (SAED) pattern, confirming the polycrystalline nature of SnO_2_ with indexed diffraction rings for the (101), (110), (211), and (220) planes, as well as a weak ring assigned to CuO (110), indicating successful Cu incorporation.


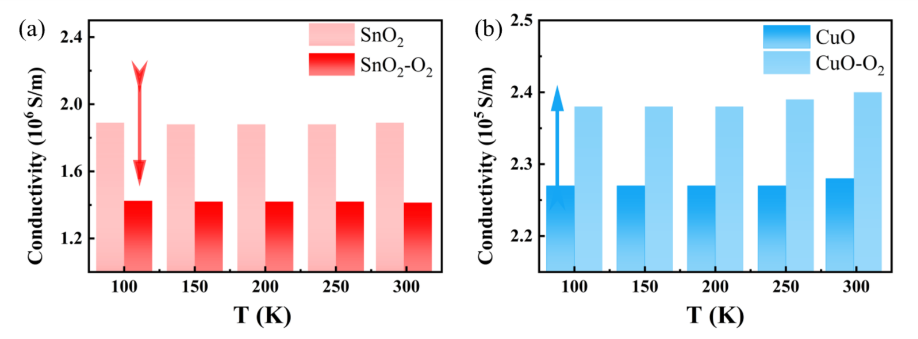


**Figure S4 Temperature-dependent simulation electrical conductivity by semi-empirical Boltzmann transport theory-based calculations.** Pristine and oxygen adsorption conductivity of (a) SnO_2_ and (b) CuO from 100 K to 300 K under vacuum.


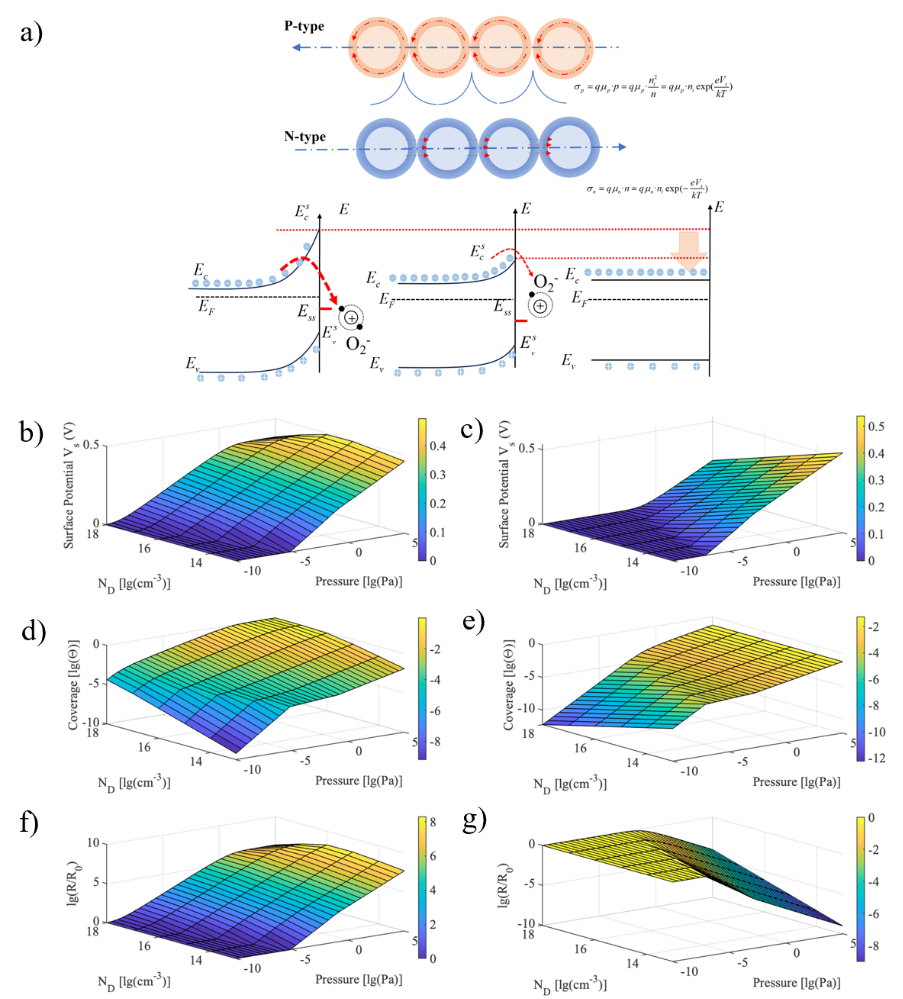


**Figure S5. Pressure-dependent physical property variations of p- and n-type semiconductors by numerical simulation.** (a) Schematic illustration of charge transport mechanisms; The surface potential (*Vₛ*), donor concentration (*N_D_*), and pressure (*P*) (b)SnO_2_ and (c)CuO; Surface coverage (*θ*) as a function of *N_D_* and *P* (d)SnO_2_ and (e)CuO; Relative resistance change (R/R_0_) versus *N_D_*  and pressure (f) SnO_2_ and (g) CuO.


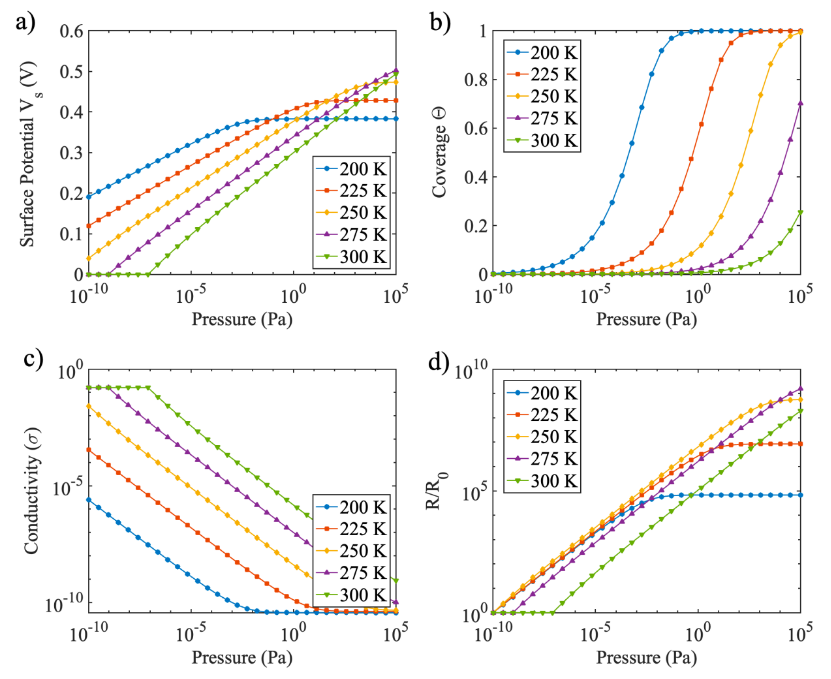


**Figure S6. Effect of pressure on n-type semiconductors (SnO_2_) at different temperatures based on numerical simulation.** (a) Surface potential (*Vₛ*) as a function of pressure (P); (b) Surface coverage (*θ*) versus pressure; (c Electrical conductivity (*σ*) as a function of pressure; (d) Relative resistance change (R/R₀) with varying pressure.


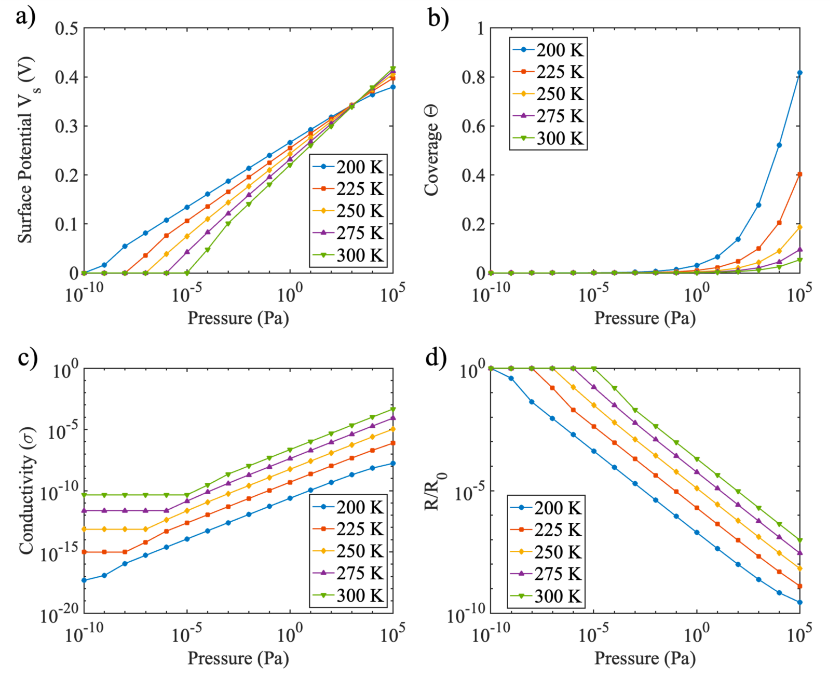


**Figure S7. Effect of pressure on p-type semiconductors (CuO) at different temperatures based on numerical simulation.** (a) Surface potential (*Vₛ*) as a function of pressure (P); (b) Surface coverage (*θ*) versus pressure; (c) Electrical conductivity (*σ*) as a function of pressure; (d) Relative resistance change (R/R₀) with varying pressure.


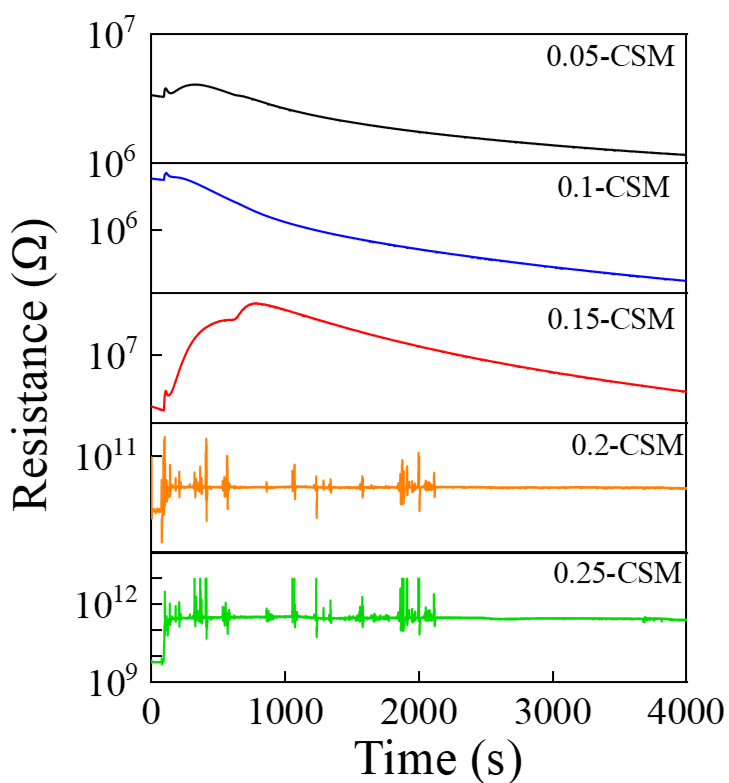


**Figure S8.** **Resistance variation of CSM-550 samples with different Cu/Sn molar ratios under dynamic vacuum pumping conditions from 10^5^ Pa to 3×10^-3^ Pa.**


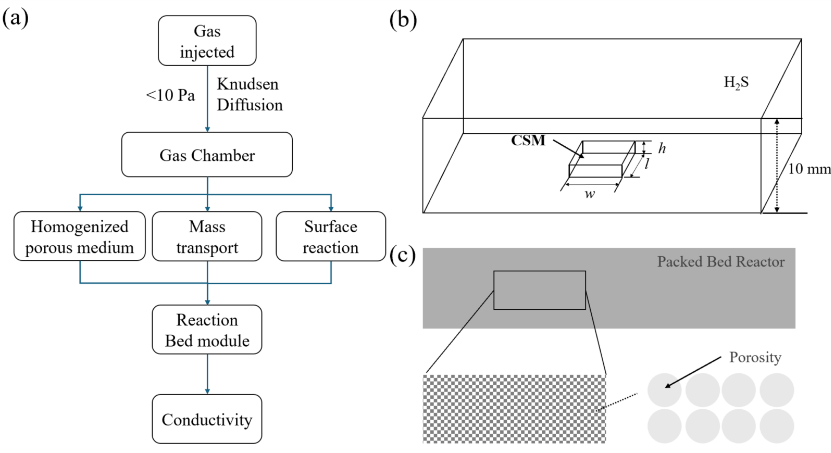


**FigureS9. The COMSOL model of CSM gas sensing in H_2_S.** (a) Scheme of process flow for model design. (b) Geometry of the gas chamber with the sensing film (CSM) exposed to H_2_S. (c) Packed bed reactor with a porous structure facilitating gas-solid interactions.


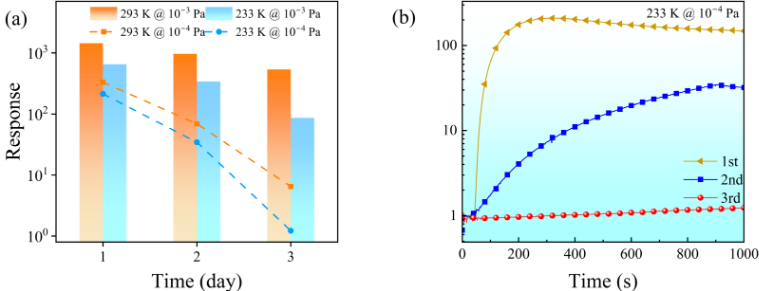


**FigureS10. Unrecovered properties of CSM-650 gas sensor under low temperature.** (a)The film was repeated at 60 ℃ for 12 h for heating after reaction with 1 ppm_a_ H_2_S under atmosphere. (b)The film was repeated at 100 ℃ for 12 h heating after reaction with 0.25 ppm_a_ H_2_S.


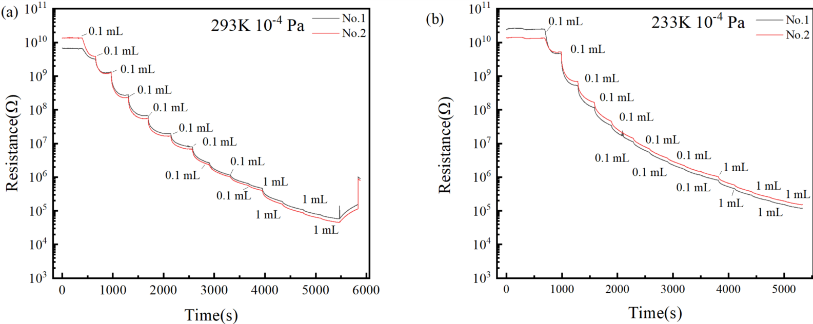


**Figure S11. Response of CSM-650 upon exposure to H_2_S (1% initial) under vacuum conditions (1 × 10⁻^4^ Pa) for** (a) 293K and (b) 233K. The injection of 0.1 mL and 1.0 mL corresponds to atmospheric-equivalent concentrations of 0.25 ppm_a_ and 2.5 ppm_a_, respectively.


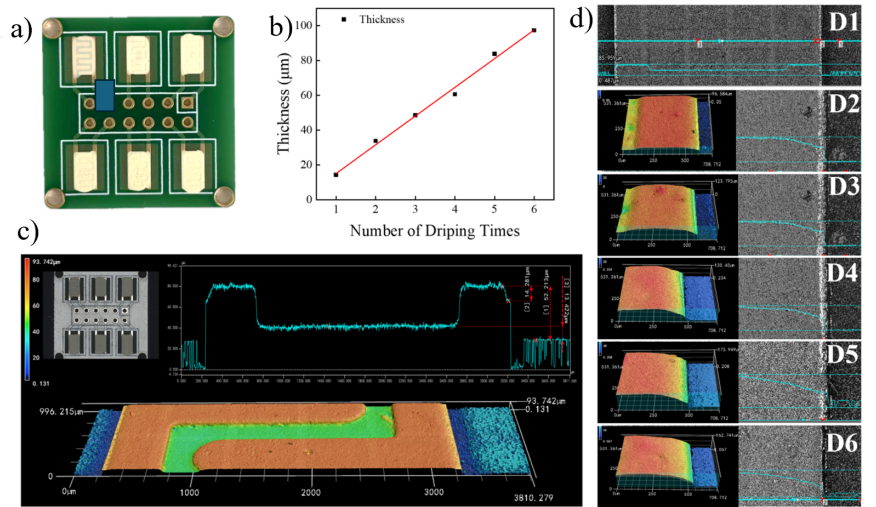


**FigureS12. Fabricated printed circuit board (PCB) and different thickness of CSM gas sensor.** (**A**) The well-defined circuitry PCB with 1 μm-thick gold (Au) layer; (**B**) The thickness of CSM films different with drop-casting cycles; (**C**) 3D profilometry results displaying the film thickness distribution and surface morphology; (**D**)Cross-sectional views and thickness profiles of films corresponding to 1 to 6 dripping times(D1–D6).
